# Ethyl 1-oxo-1,2,3,4-tetra­hydro-9*H*-carbazole-3-carboxyl­ate

**DOI:** 10.1107/S160053680902385X

**Published:** 2009-06-27

**Authors:** Tuncer Hökelek, Hakan Dal, Barış Tercan, Mustafa Göçmentürk, Yavuz Ergün

**Affiliations:** a Department of Physics, Hacettepe University, 06800 Beytepe, Ankara, Turkey; b Department of Chemistry, Faculty of Science, Anadolu University, 26470 Yenibağlar, Eskişehir, Turkey; c Department of Physics, Karabük University, 78050 Karabük, Turkey; d Department of Chemistry, Faculty of Arts and Sciences, Dokuz Eylül University, Tınaztepe, 35160 Buca-zmir, Turkey

## Abstract

The title compound, C_15_H_15_NO_3_, contains a carbazole skeleton with an ethoxy­carbonyl group at the 3 position. In the indole ring system, the benzene and pyrrole rings are nearly coplanar, forming a dihedral angle of 1.95 (8)°. The cyclo­hexenone ring has an envelope conformation. In the crystal structure, pairs of strong N—H⋯O hydrogen bonds link the mol­ecules into centrosymmetric dimers with *R* ^2^_2_(10) ring motifs. π–π contacts between parallel pyrrole rings [centroid–centroid distance = 3.776 (2) Å] may further stabilize the structure. A weak C—H⋯π inter­action is also observed.

## Related literature

For tetrahydrocarbazole derivatives as synthetic precursors of cyclic indole-type alkaloids of biological interest, see: Abraham (1975 ▶); Phillipson & Zenk (1980 ▶); Saxton (1983 ▶).  The title compound is used in the synthesis of a precursor for the synthesis of the anti-tumor drug ellipticine (Ergün *et al.*, 2004 ▶). Murraya *L*. (Rutaceae) is a genus of shrubs or small trees from Southern Asia (Chang, 1977 ▶) from which carbazole alkaloids have been isolated (Chakraborty & Roy, 1991 ▶). For the biological activity of carbazole alkaloids, see: Kondo *et al.* (1986 ▶); Te Paske *et al.* (1989*a* ▶,*b* ▶). For related structures, see: Çaylak *et al.* (2007 ▶); Uludağ *et al.* (2009 ▶). For bond-length data, see: Allen *et al.* (1987 ▶). For ring-motifs, see: Bernstein *et al.* (1995 ▶).
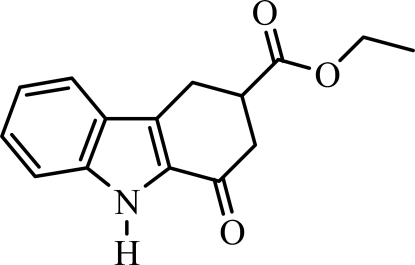


## Experimental

### 

#### Crystal data

C_15_H_15_NO_3_
*M* *_r_* = 257.28
Monoclinic, 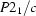

*a* = 5.6811 (3) Å
*b* = 8.7378 (5) Å
*c* = 24.8310 (14) Å
β = 93.208 (4)°
*V* = 1230.69 (12) Å^3^
*Z* = 4
Mo *K*α radiation
μ = 0.10 mm^−1^
*T* = 100 K
0.45 × 0.20 × 0.15 mm

#### Data collection

Bruker Kappa APEXII CCD area-detector diffractometer
Absorption correction: multi-scan (*SADABS*; Bruker, 2005 ▶) *T*_min_ = 0.958, *T*_max_ = 0.983
9148 measured reflections
3004 independent reflections
2145 reflections with *I* > 2σ(*I*)
*R*_int_ = 0.059

#### Refinement

*R*[*F* ^2^ > 2σ(*F* ^2^)] = 0.075
*wR*(*F* ^2^) = 0.221
*S* = 1.05
3004 reflections
177 parameters
H atoms treated by a mixture of independent and constrained refinement
Δρ_max_ = 1.25 e Å^−3^
Δρ_min_ = −0.42 e Å^−3^

### 

Data collection: *APEX2* (Bruker, 2007 ▶); cell refinement: *SAINT* (Bruker, 2007 ▶); data reduction: *SAINT*; program(s) used to solve structure: *SHELXS97* (Sheldrick, 2008 ▶); program(s) used to refine structure: *SHELXL97* (Sheldrick, 2008 ▶); molecular graphics: *ORTEP-3 for Windows* (Farrugia, 1997 ▶) and Mercury (Macrae *et al.*, 2006 ▶); software used to prepare material for publication: *WinGX* (Farrugia, 1999 ▶) and *PLATON* (Spek, 2009 ▶).

## Supplementary Material

Crystal structure: contains datablocks I, global. DOI: 10.1107/S160053680902385X/xu2543sup1.cif

Structure factors: contains datablocks I. DOI: 10.1107/S160053680902385X/xu2543Isup2.hkl

Additional supplementary materials:  crystallographic information; 3D view; checkCIF report

## Figures and Tables

**Table 1:** Hydrogen-bond geometry (Å, °)

*D*—H⋯*A*	*D*—H	H⋯*A*	*D*⋯*A*	*D*—H⋯*A*
N9—H9⋯O1^i^	0.82 (4)	2.08 (4)	2.834 (3)	154 (4)
C4—H4*A*⋯*Cg*3^ii^	0.99	2.75	3.727 (3)	171

Symmetry codes: (i) 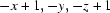; (ii) 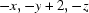. *Cg*3 is the centroid of the C5*A*/C5–C8/C8*A* ring.

**Fig. 1.**
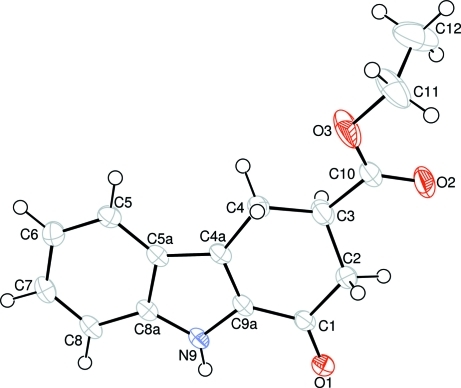
The molecular structure of the title molecule with the atom-numbering scheme. The displacement ellipsoids are drawn at the 50% probability level.

**Fig. 2.**
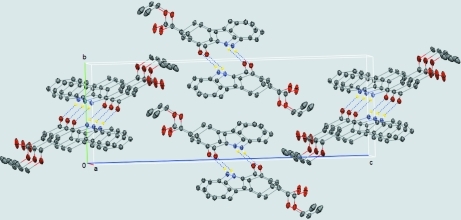
A partial packing diagram for the title compound. Hydrogen bonds are shown as dashed lines. H atoms not involved in hydrogen bonding have been omitted for clarity.

## supplementary crystallographic information

### Comment

Tetrahydrocarbazole derivatives can be considered to be synthetic precursors of cyclic indole-type alkaloids of biological interest (Abraham, 1975; Phillipson & Zenk, 1980; Saxton, 1983). The title compound was used in the synthesis of the precursor compound for the synthesis of anti-tumor drug ellipticine (Ergün *et al.*, 2004). Murraya *L*. (Rutaceae) is a genus of shrubs or small trees from Southern Asia (Chang, 1977). The main constituent of this genus include carbazole alkaloids (Chakraborty & Roy, 1991). Several biological properties have been reported for carbazole alkaloids including antibiotic, cytotoxic and antiviral activities (Kondo *et al.*, 1986; Te Paske *et al.*, 1989*a*,b). The title compound may also be used as a precursor in the synthesis of Murraya alkaloids. The present study was undertaken to ascertain its crystal structure.

The molecule of the title compound (Fig. 1) contains a carbazole skeleton with a carboxyethyl group at position 3, where the bond lengths (Allen *et al.*, 1987) and angles are within normal ranges.

An examination of the deviations from the least-squares planes through individual rings shows that rings B (C4a/C5a/C8a/N9/C9a) and C (C5a/C5—C8/C8a) are nearly coplanar [with a maximum deviation of -0.028 (3) Å for atom C4a] with dihedral angle of A/B = 1.95 (8)°. Ring A (C1—C4/C4a/C9a) adopts envelope conformation with atom C3 displaced by 0.527 (3) Å from the plane of the other rings atoms, as in 3a,4,10,10*b*-tetrahydro-2*H*-furo[2,3-*a*]carbazol-5(3*H*)-one (Çaylak *et al.*, 2007) and 3,3-ethylenedithio-3,3a,4,5,10,10*b*-hexahydro-2*H*-furo[2,3-*a*]carbazole (Uludağ *et al.*, 2009).

In the crystal structure, pairs of strong intermolecular N—H···O hydrogen bonds (Table 1) link the molecules into centosymmetric dimers with *R*^2^_2_(10) ring motifs (Bernstein *et al.*, 1995) (Fig. 2), in which they may be effective in the stabilization of the structure. The π–π contact between the pyrrole rings, *Cg*2—*Cg*2^i^, [symmetry code:(i) -*x*, 1 - *y*, -*z*, where *Cg*2 is centroid of the ring B (C4a/C5a/C8a/N9/C9a)] may further stabilize the structure, with centroid-centroid distance of 3.776 (2) Å. There also exists a weak C—H···π interaction (Table 1).

### Experimental

For the preparation of the title compound, a solution of ethyl 1,2,3,4-tetrahydro-9*H*-carbazole-3-carboxylate (5.00 g, 20.5 mmol) in methanol (25 ml) was added dropwise to a solution of periodic acid (9.35 g, 41.0 mmol) in methanol-water (1:1, 100 ml) at 273 K. The reaction mixture was stirred for 1 h at 273 K, then stirring was continued for a further 1 h at room temperature. The solvent was evaporated, then the residue was dissolved in chloroform and washed first with sodium carbonate (10%, 50 ml) and then with sodium bisulfite (10%, 50 ml). The organic layer was dried over anhydrous magnesium sulfate and the solvent was evaporated. The residue was chromatographed on silica gel using ethyl acetate and crystallized from methanol (yield; 3.17 g, 67%, m.p. 411 K).

### Refinement

The highest peak in the final difference electron-density map is apart 0.94 Å from atom C3. Atom H9 (for NH) was located in difference Fourier map and refined isotropically. The remaining H atoms were positioned geometrically, with C—H = 0.95, 1.00, 0.99 and 0.98 Å for aromatic, methine, methylene and methyl H, respectively, and constrained to ride on their parent atoms, with *U*_iso_(H) = *xU*_eq_(C), where *x* = 1.5 for methyl H and *x* = 1.2 for all other H atoms.

### Crystal data

**Table:**

C_15_H_15_NO_3_	*F*(000) = 544
*M**_r_* = 257.28	*D*_x_ = 1.389 Mg m^−^^3^
Monoclinic, *P*2_1_/*c*	Mo *K*α radiation, λ = 0.71073 Å
Hall symbol: -P 2ybc	Cell parameters from 3480 reflections
*a* = 5.6811 (3) Å	θ = 1.6–28.3°
*b* = 8.7378 (5) Å	µ = 0.10 mm^−^^1^
*c* = 24.8310 (14) Å	*T* = 100 K
β = 93.208 (4)°	Rod-shaped, colorless
*V* = 1230.69 (12) Å^3^	0.45 × 0.20 × 0.15 mm
*Z* = 4	

### Data collection

**Table:**

Bruker Kappa APEXII CCD area-detector diffractometer	3004 independent reflections
Radiation source: fine-focus sealed tube	2145 reflections with *I* > 2σ(*I*)
graphite	*R*_int_ = 0.059
φ and ω scans	θ_max_ = 28.3°, θ_min_ = 1.6°
Absorption correction: multi-scan (*SADABS*; Bruker, 2005)	*h* = −7→7
*T*_min_ = 0.958, *T*_max_ = 0.983	*k* = −11→9
9148 measured reflections	*l* = −32→28

### Refinement

**Table:**

Refinement on *F*^2^	Primary atom site location: structure-invariant direct methods
Least-squares matrix: full	Secondary atom site location: difference Fourier map
*R*[*F*^2^ > 2σ(*F*^2^)] = 0.075	Hydrogen site location: inferred from neighbouring sites
*wR*(*F*^2^) = 0.221	H atoms treated by a mixture of independent and constrained refinement
*S* = 1.05	*w* = 1/[σ^2^(*F*_o_^2^) + (0.1083*P*)^2^ + 1.2571*P*] where *P* = (*F*_o_^2^ + 2*F*_c_^2^)/3
3004 reflections	(Δ/σ)_max_ < 0.001
177 parameters	Δρ_max_ = 1.25 e Å^−^^3^
0 restraints	Δρ_min_ = −0.42 e Å^−^^3^

### Special details

**Table:**

Geometry. All e.s.d.'s (except the e.s.d. in the dihedral angle between two l.s. planes) are estimated using the full covariance matrix. The cell e.s.d.'s are taken into account individually in the estimation of e.s.d.'s in distances, angles and torsion angles; correlations between e.s.d.'s in cell parameters are only used when they are defined by crystal symmetry. An approximate (isotropic) treatment of cell e.s.d.'s is used for estimating e.s.d.'s involving l.s. planes.
Refinement. Refinement of *F*^2^ against ALL reflections. The weighted *R*-factor *wR* and goodness of fit *S* are based on *F*^2^, conventional *R*-factors *R* are based on *F*, with *F* set to zero for negative *F*^2^. The threshold expression of *F*^2^ > σ(*F*^2^) is used only for calculating *R*-factors(gt) *etc*. and is not relevant to the choice of reflections for refinement. *R*-factors based on *F*^2^ are statistically about twice as large as those based on *F*, and *R*- factors based on ALL data will be even larger.

### Fractional atomic coordinates and isotropic or equivalent isotropic displacement parameters (Å^2^)

**Table:**

	*x*	*y*	*z*	*U*_iso_*/*U*_eq_	
O1	0.4546 (3)	0.0609 (2)	0.42291 (7)	0.0294 (5)	
O2	−0.0619 (5)	0.3051 (4)	0.27554 (9)	0.0775 (11)	
O3	−0.2959 (5)	0.4520 (3)	0.32045 (8)	0.0603 (8)	
C1	0.2768 (4)	0.1407 (3)	0.42209 (10)	0.0245 (5)	
C2	0.1783 (5)	0.2133 (4)	0.37051 (10)	0.0316 (6)	
H2A	0.1988	0.1406	0.3405	0.038*	
H2B	0.2727	0.3056	0.3634	0.038*	
C3	−0.0831 (5)	0.2597 (4)	0.36991 (11)	0.0355 (7)	
H3	−0.1758	0.1623	0.3689	0.043*	
C4	−0.1525 (4)	0.3454 (3)	0.41897 (10)	0.0263 (6)	
H4A	−0.0978	0.4527	0.4171	0.032*	
H4B	−0.3264	0.3461	0.4202	0.032*	
C4A	−0.0453 (4)	0.2708 (3)	0.46893 (10)	0.0229 (5)	
C5	−0.2963 (4)	0.3489 (3)	0.54916 (10)	0.0270 (6)	
H5	−0.4152	0.4041	0.5289	0.032*	
C5A	−0.1107 (4)	0.2805 (3)	0.52332 (9)	0.0232 (5)	
C6	−0.3046 (5)	0.3353 (3)	0.60418 (11)	0.0313 (6)	
H6	−0.4315	0.3802	0.6219	0.038*	
C7	−0.1266 (5)	0.2553 (3)	0.63473 (10)	0.0314 (6)	
H7	−0.1334	0.2502	0.6728	0.038*	
C8	0.0550 (5)	0.1850 (3)	0.61069 (10)	0.0283 (6)	
H8	0.1737	0.1313	0.6315	0.034*	
C8A	0.0608 (4)	0.1948 (3)	0.55452 (10)	0.0243 (5)	
N9	0.2174 (4)	0.1343 (3)	0.52100 (8)	0.0240 (5)	
H9	0.335 (7)	0.082 (4)	0.5280 (15)	0.050 (11)*	
C9A	0.1539 (4)	0.1807 (3)	0.46917 (9)	0.0233 (5)	
C10	−0.1422 (5)	0.3404 (4)	0.31657 (11)	0.0343 (7)	
C11	−0.3501 (8)	0.5424 (4)	0.27171 (14)	0.0588 (11)	
H11A	−0.3926	0.6478	0.2821	0.071*	
H11B	−0.2082	0.5483	0.2504	0.071*	
C12	−0.5423 (7)	0.4765 (6)	0.23878 (18)	0.0713 (13)	
H12A	−0.5770	0.5416	0.2072	0.107*	
H12B	−0.6825	0.4693	0.2599	0.107*	
H12C	−0.4976	0.3741	0.2269	0.107*	

### Atomic displacement parameters (Å^2^)

**Table:**

	*U*^11^	*U*^22^	*U*^33^	*U*^12^	*U*^13^	*U*^23^
O1	0.0278 (9)	0.0346 (11)	0.0253 (9)	0.0038 (8)	−0.0018 (7)	0.0018 (8)
O2	0.0752 (18)	0.139 (3)	0.0185 (10)	0.0649 (19)	0.0037 (11)	0.0088 (13)
O3	0.103 (2)	0.0541 (16)	0.0224 (10)	0.0415 (15)	−0.0067 (11)	0.0049 (10)
C1	0.0240 (11)	0.0279 (14)	0.0213 (11)	−0.0035 (10)	−0.0028 (9)	0.0015 (10)
C2	0.0282 (13)	0.0451 (18)	0.0215 (12)	0.0037 (12)	0.0013 (10)	0.0077 (11)
C3	0.0433 (16)	0.0393 (18)	0.0234 (13)	0.0103 (13)	−0.0034 (11)	−0.0001 (11)
C4	0.0259 (12)	0.0319 (15)	0.0211 (11)	0.0004 (11)	0.0000 (9)	0.0074 (10)
C4A	0.0244 (11)	0.0230 (13)	0.0209 (11)	−0.0034 (10)	−0.0032 (9)	0.0035 (9)
C5	0.0274 (12)	0.0279 (14)	0.0254 (12)	−0.0009 (10)	−0.0016 (9)	0.0029 (10)
C5A	0.0267 (12)	0.0223 (13)	0.0200 (11)	−0.0048 (10)	−0.0037 (9)	0.0025 (9)
C6	0.0334 (13)	0.0349 (16)	0.0259 (13)	0.0017 (12)	0.0033 (10)	−0.0001 (11)
C7	0.0415 (15)	0.0333 (16)	0.0193 (11)	−0.0027 (12)	−0.0009 (10)	0.0013 (11)
C8	0.0353 (13)	0.0285 (15)	0.0203 (12)	−0.0019 (11)	−0.0062 (10)	0.0020 (10)
C8A	0.0268 (12)	0.0240 (13)	0.0213 (11)	−0.0033 (10)	−0.0040 (9)	0.0007 (10)
N9	0.0278 (11)	0.0250 (12)	0.0186 (10)	0.0007 (9)	−0.0043 (8)	0.0003 (8)
C9A	0.0274 (12)	0.0237 (13)	0.0183 (11)	−0.0049 (10)	−0.0035 (9)	0.0024 (9)
C10	0.0376 (14)	0.0441 (18)	0.0207 (12)	0.0060 (13)	−0.0035 (10)	0.0014 (11)
C11	0.101 (3)	0.040 (2)	0.0337 (17)	0.013 (2)	−0.0184 (18)	0.0118 (14)
C12	0.053 (2)	0.083 (3)	0.076 (3)	−0.005 (2)	−0.022 (2)	0.041 (2)

### Geometric parameters (Å, °)

**Table:**

O1—C1	1.227 (3)	C6—C5	1.375 (4)
O2—C10	1.180 (3)	C6—H6	0.9500
O3—C10	1.315 (4)	C7—C6	1.415 (4)
O3—C11	1.464 (4)	C7—C8	1.366 (4)
C1—C9A	1.438 (3)	C7—H7	0.9500
C2—C1	1.508 (3)	C8—C8A	1.400 (3)
C2—H2A	0.9900	C8—H8	0.9500
C2—H2B	0.9900	C8A—N9	1.359 (3)
C3—C2	1.539 (4)	C8A—C5A	1.423 (3)
C3—C4	1.501 (4)	N9—H9	0.82 (4)
C3—C10	1.521 (4)	C9A—C4A	1.378 (4)
C3—H3	1.0000	C9A—N9	1.378 (3)
C4—C4A	1.500 (3)	C11—C12	1.446 (6)
C4—H4A	0.9900	C11—H11A	0.9900
C4—H4B	0.9900	C11—H11B	0.9900
C5—C5A	1.399 (4)	C12—H12A	0.9800
C5—H5	0.9500	C12—H12B	0.9800
C5A—C4A	1.423 (3)	C12—H12C	0.9800
			
C10—O3—C11	116.7 (3)	C5—C6—H6	119.6
O1—C1—C2	121.3 (2)	C7—C6—H6	119.6
O1—C1—C9A	124.2 (2)	C6—C7—H7	119.3
C9A—C1—C2	114.5 (2)	C8—C7—C6	121.5 (2)
C1—C2—C3	115.5 (2)	C8—C7—H7	119.3
C1—C2—H2A	108.4	C7—C8—C8A	118.0 (2)
C1—C2—H2B	108.4	C7—C8—H8	121.0
C3—C2—H2A	108.4	C8A—C8—H8	121.0
C3—C2—H2B	108.4	N9—C8A—C5A	108.9 (2)
H2A—C2—H2B	107.5	N9—C8A—C8	129.8 (2)
C2—C3—H3	106.4	C8—C8A—C5A	121.4 (2)
C4—C3—C2	114.9 (2)	C8A—N9—C9A	108.1 (2)
C4—C3—C10	114.8 (2)	C8A—N9—H9	130 (3)
C4—C3—H3	106.4	C9A—N9—H9	122 (3)
C10—C3—C2	107.3 (2)	N9—C9A—C1	125.0 (2)
C10—C3—H3	106.4	C4A—C9A—N9	110.1 (2)
C3—C4—H4A	109.7	C4A—C9A—C1	124.8 (2)
C3—C4—H4B	109.7	O2—C10—O3	123.2 (3)
C4A—C4—C3	110.0 (2)	O2—C10—C3	123.6 (3)
C4A—C4—H4A	109.7	O3—C10—C3	113.3 (2)
C4A—C4—H4B	109.7	O3—C11—H11A	109.3
H4A—C4—H4B	108.2	O3—C11—H11B	109.3
C5A—C4A—C4	130.1 (2)	C12—C11—O3	111.7 (3)
C9A—C4A—C4	123.2 (2)	C12—C11—H11A	109.3
C9A—C4A—C5A	106.7 (2)	C12—C11—H11B	109.3
C5A—C5—H5	120.4	H11A—C11—H11B	107.9
C6—C5—C5A	119.2 (2)	C11—C12—H12A	109.5
C6—C5—H5	120.4	C11—C12—H12B	109.5
C4A—C5A—C8A	106.1 (2)	C11—C12—H12C	109.5
C5—C5A—C4A	134.8 (2)	H12A—C12—H12B	109.5
C5—C5A—C8A	119.1 (2)	H12A—C12—H12C	109.5
C5—C6—C7	120.8 (3)	H12B—C12—H12C	109.5
			
C11—O3—C10—O2	−5.3 (5)	C5—C5A—C4A—C4	5.2 (5)
C11—O3—C10—C3	175.9 (3)	C5—C5A—C4A—C9A	−177.5 (3)
C10—O3—C11—C12	88.6 (5)	C8A—C5A—C4A—C4	−176.3 (3)
O1—C1—C9A—N9	−0.6 (4)	C8A—C5A—C4A—C9A	1.0 (3)
O1—C1—C9A—C4A	179.6 (2)	C7—C6—C5—C5A	0.9 (4)
C2—C1—C9A—N9	−176.9 (2)	C8—C7—C6—C5	−2.0 (4)
C2—C1—C9A—C4A	3.2 (4)	C6—C7—C8—C8A	0.2 (4)
C3—C2—C1—O1	158.9 (3)	C7—C8—C8A—N9	−178.9 (3)
C3—C2—C1—C9A	−24.5 (4)	C7—C8—C8A—C5A	2.7 (4)
C4—C3—C2—C1	46.9 (4)	N9—C8A—C5A—C4A	−1.2 (3)
C10—C3—C2—C1	175.9 (2)	N9—C8A—C5A—C5	177.5 (2)
C2—C3—C4—C4A	−44.0 (3)	C8—C8A—C5A—C4A	177.5 (2)
C10—C3—C4—C4A	−169.1 (2)	C8—C8A—C5A—C5	−3.7 (4)
C2—C3—C10—O2	36.0 (5)	C8—C8A—N9—C9A	−177.6 (3)
C2—C3—C10—O3	−145.3 (3)	C5A—C8A—N9—C9A	1.0 (3)
C4—C3—C10—O2	165.0 (3)	C1—C9A—N9—C8A	179.8 (2)
C4—C3—C10—O3	−16.3 (4)	C4A—C9A—N9—C8A	−0.4 (3)
C3—C4—C4A—C5A	−159.6 (3)	N9—C9A—C4A—C4	177.1 (2)
C3—C4—C4A—C9A	23.5 (4)	N9—C9A—C4A—C5A	−0.4 (3)
C6—C5—C5A—C4A	−179.8 (3)	C1—C9A—C4A—C4	−3.1 (4)
C6—C5—C5A—C8A	1.8 (4)	C1—C9A—C4A—C5A	179.4 (2)

### Hydrogen-bond geometry (Å, °)

**Table:**

*D*—H···*A*	*D*—H	H···*A*	*D*···*A*	*D*—H···*A*
N9—H9···O1^i^	0.82 (4)	2.08 (4)	2.834 (3)	154 (4)
C4—H4A···Cg3^ii^	0.99	2.75	3.727 (3)	171

Symmetry codes: (i) −*x*+1, −*y*, −*z*+1; (ii) −*x*, −*y*+2, −*z*.
